# Influences of Serotonin Hydrochloride on *Adiponectin*, *Ghrelin* and *KiSS1* Genes Expression

**DOI:** 10.31661/gmj.v9i0.1767

**Published:** 2020-09-07

**Authors:** Fariba Mahmoudi, Khadijeh Haghighat Gollo

**Affiliations:** ^1^Faculty of Sciences, University of Mohaghegh Ardabili, Ardabil, Iran

**Keywords:** Serotonin, Kisspeptin, Ghrelin, Adiponectin

## Abstract

**Background:**

Serotonin and kisspeptin stimulates gonadotropin-releasing hormone (GnRH) and luteinizing hormone (LH) release while ghrelin and adiponectin inhibit them. In the present experimental study, the effects of central injection of serotonin were investigated on LH concentration, *KiSS1*, *adiponectin*, and *ghrelin* genes expression.

**Materials and Methods:**

Fifteen Wistar male rats in three groups received saline or serotonin hydrochloride via the third cerebral ventricle. Blood samples were collected via the tail vein. Serum LH concentration and relative gene expression were evaluated by radioimmunoassay and real-time polymerase chain reaction method, respectively.

**Results:**

Serotonin significantly increased the mean serum LH concentration and *KiSS1* gene expression levels compared to the saline group. Serotonin significantly decreased the mean *ghrelin* and *adiponectin* genes expression levels compared to the saline group.

**Conclusion:**

The serotonergic pathway may have stimulatory effects on hypothalamic kisspeptin synthesis, partly via inhibiting hypothalamic ghrelin and adiponectin neural activity.

## Introduction

Several hypothalamic neuronal circuits act together to control the hypothalamus-pituitary- gonadal (HPG) axis. Serotonin is a neurotransmitter that is synthesized from tryptophan amino acid by the action of tryptophan hydroxylase and aromatic amino acid decarboxylase enzymes [1]. In the brain, serotonin is synthesized mainly in the raphe nucleus of the brainstem and from this site, its neural axons project to most parts of the brain, e.g., the hypothalamus [1-2]. Also, in the peripheral organs, serotonin and its receptors are expressed in the gut and gonads [3-4]. In addition to the regulation of food intakes, serotonin stimulates gonadotropin-releasing hormone (GnRH) and luteinizing hormone (LH) release [5-6]. Kisspeptin is a 54 amino acid neuropeptide that is coded by of *KiSS1* gene and shorter products, including kisspeptin-14, 13, and 10 proteolytically cleave of it. All kisspeptins exert their physiological functions via binding to G protein-coupled receptor, GPR54 [7-9]. Kisspeptin and its receptor are widely distributed in the hypothalamic nuclei involved in regulating reproduction, including the arcuate nucleus (ARC) and medial preoptic area (mPOA) [7-9]. GPR54 is extensively expressed on GnRH neurons, and kisspeptin injection is associated with strong stimulation of GnRH/LH release [9-10]. Adiponectin, a 244 amino acid peptide, is synthesized in several tissues, including the adipose tissue, hypothalamus, and gonads [11]. The physiological actions of it are mediated by two types of receptors named adiponectin receptor 1 (AdipoR_1_) and 2 (AdipoR_2_), which are extensively expressed in the lateral and medial hypothalamus gonads. It takes part in the regulation of energy homeostasis, food intakes, cardiovascular protection, and it inhibits GnRH/LH secretion [12]. There is a close relationship between metabolic and reproductive disorders and plasma adiponectin levels [13]. Ghrelin is a 28 amino acid peptide that is secreted by the stomach and central nervous system, specially hypothalamic nuclei involved in controlling energy balance and reproduction [14-15]. It stimulates food intakes and growth hormone secretion via binding to growth hormone secretagogues receptor (GHSR-Ia) [14-15]. It inhibits GnRH/LH hormone secretion) [14-15]. Due to the interference of serotonin, ghrelin, adiponectin, and kisspeptin in the endocrine control of LH secretion, in the present study, the effects of serotonin were investigated on serum LH concentration and the relative gene expression of *ghrelin, adiponectin*, and * KiSS1* in the hypothalamus of male rats.

## Materials and Methods

 In the present experimental study, male Wistar rats were housed in the cages under controlled temperature and light (12h light/ dark cycle). Animals had free access to food and water all the time. Following anesthetization by a mixture of ketamine and xylazine (ketamine 80 mg/kg + xylazine 10 mg/ kg), a 22- gauge stainless cannulae was implanted into third cerebral ventricle coordinates (AP=- 2.3, ML=0.0, DV=6.5). After surgery, animals were kept in individual cages for one week. Then fifteen rats in three groups received saline (3µl) or serotonin hydrochloride (10 or 20 µg/3µl). Serotonin hydrochloride (Sigma Aldrich, USA) was dissolved in distilled water, and it was injected by a 27- gauge stainless steel injector by using a hamilton micro syringe via third cerebral ventricle at 8:00- 9:00.

Blood samples were collected at 60min following injections via the tail vein. Blood samples were centrifuged to 15min at 3000 rpm, and the serum stored at –20°C until assayed for LH concentration. Mean serum LH concentration was determined by using the rat LH kit and the method of the radioimmunoassay (RIA). Following deep anesthetization by a mixture of ketamine and xylazine, hypothalamic samples were dissected according to Paxinos & Watson atlas and stored at -80°C. RNA of samples was isolated using the acid guanidinium thiocyanate-phenol-chloroform extraction method according to PureZol manufacturer instruction. The RNA concentration was determined by nanodrop spectrophotometer, and 1μg of total RNA of each sample was used for reverse transcription with Strand cDNA Synthesis Kit following the manufacturer’s protocol. The Corbett rotor gene 6000 real-time polymerase chain reaction (PCR) detection system and SYBR Green I kit were used for determination the gene expression levels. The PCR cycling conditions were as following: first denaturation 95 Cº for 2 min, followed by 40 cycles of denaturation at 95 Cº for 5 sec, annealing at 54 Cº for 20 sec ( *ghrelin*) and annealing at 60 Cº for 20 sec ( *adiponectin*, *KiSS1* or *GAPDH*) and extension at 60 Cº for 25 sec. Specific oligonucleotide sequences for forward and reverse primers used were: *KiSS1* forward 5′-TGATCTCGCTGGCTTCTTGGC-3′ and reverse 5′-GGGTTCAGGGTTCACCACAGG-3′, * adiponectin* forward: 5′-AATCCTGCCCAGTCATGAAG-3′ and reverse: 5′-CATCTCCTGGGTCACCCTTA-3′, *ghrelin* forward: 5′-AATGCTCCCTTCGAT GTTGG-3′ and reverse: 5′-CAGTGGTTACTTGTTAGCTGG-3′ and *GAPDH* forward: 5′-AAGTTCAACGGCACAGTCAAG-3′ and reverse: 5′- CATACTCAGCACCAGCATCAC-3′. The *adiponectin*, *ghrelin*, *KiSS1*, and *GAPDH *ampliﬁed products were 214, 98, 132, and 120 base pairs, respectively. The calculation of relative gene expression levels of the target mRNAs were calculated by the equation 2^-ΔΔCT^. The data were analyzed by SPSS software version 16 ( SPSS Inc. USA) One- way ANOVA test was used to analyze the gene expression and hormonal data. Post hoc Tukey test was used to compare the significant difference between control and experimental groups. The results are presented as mean ± SEM. In all cases, significance was defined by P≤0.05.

###  Ethical Issue

 All procedures for the maintenance and the use of experimental animals were executed with the Guide for the Care and Use of Laboratory Animals (National Institute of Health Publication No. 80-23, revised 1996. This research was approved by ethic committee of University of Mohaghegh Ardabili (code: 94:253).

## Results

**Figure 1:**
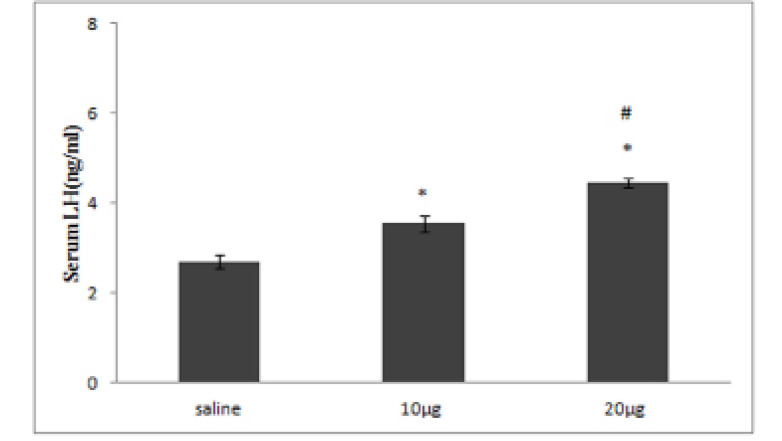
The effects of serotonin hydrocloride on mean serum LH concentration. *: compared to saline, #: compared to 10µg serotonin (data presented as mean ± SEM, P≤0.05, n=5 in each group).

**Figure 2:**
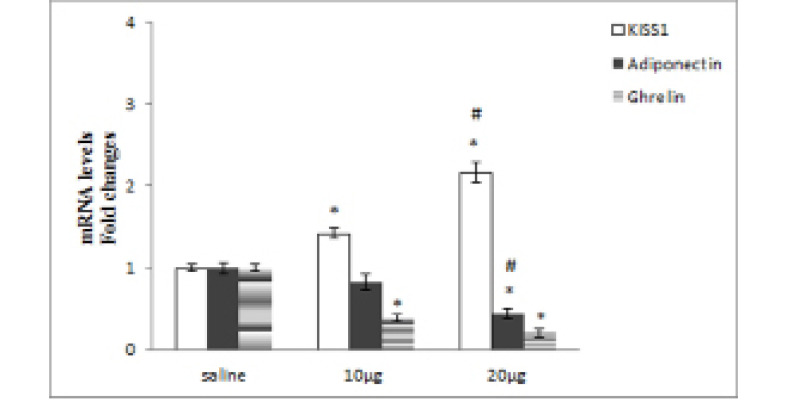
The effects of serotonin hydrocloride on mean gene expression levels of KiSS1, adiponectin and ghrelin. *: compared to saline, **#:** compared to 10µg serotonin (data presented as mean ± SEM, P≤0.05, n=5 in each group).

Injections of 10 or 20µg serotonin significantly increased the mean serum LH concentrations by 0.31 or 0.65 times compared to saline, respectively (P≤0.05, Figure-1). Injections of 20µg serotonin significantly increased the mean serum LH concentration by 0.25 compared to the 10µg serotonin group (P≤0.05, Figure-1). Injections of 10 or 20µg of serotonin significantly increased the mean *KiSS1* gene expression levels by 0.42 or 1.17 times in comparison to the saline group, respectively (P≤0.05, Figure-2). Injections of 20µg serotonin significantly increased the mean *KiSS1* gene expression levels by 0.52 compared to 10µg serotonin group (P≤0.05, Figure-2). Mean *adiponectin* gene expression levels decreased by 0.17 or 0.56 times following injections of 10 or 20µg serotonin compared to saline. This decrease in 10µg serotonin group was not statistically significant, but in 20µg serotonin group was statistically significant in comparison to the saline group (P≤0.05, Figure-2). Injections of 20µg serotonin significantly decreased the mean *adiponectin* gene expression levels by 0.39 compared to 10µg serotonin receiving rats (P≤0.05, Figure-2). Infusion of 10 or 20µg serotonin significantly decreased the mean *ghrelin* gene expression levels by 0.61 or 0.79 times compared to the saline group, respectively (P≤0.05, Figure-2). Injections of 20µg serotonin decreased the mean *ghrelin* gene expression levels by 0.46 compared to 10µg serotonin receiving rats, but this decrease was not statistically significant (Figure-2).

## Discussion

The results of the present study showed that the mean serum LH concentration significantly increased in serotonin receiving rats in comparison to the saline group. The present data are in accordance with previous studies that established the stimulatory influences of the serotonergic pathway on controlling GnRH/LH release [5-6]. It is completely established that the kisspeptin/GPR54 signaling pathway is an important stimulatory neuronal pathway upstream GnRH neurons for controlling LH secretion [7-10]. We investigated the effects of third cerebral ventricular injections of serotonin on *KiSS1* gene expression in vivo. The results demonstrated the stimulatory effects of serotonin on *KiSS1* mRNA levels compared to saline receiving rats. The present data are in agreement with the previous studies, which demonstrated an interaction between serotoninergic and kisspeptin signaling systems. In zebrafish, a relationship has been shown between kisspeptin and serotonergic signaling system. So that kisspeptin neurons modulate the raphe nucleus serotonergic neuron activity [16]. To find some mechanisms involved in the regulation of kisspeptin synthesis by serotonin, we try to investigate the effects of serotonin on gene expression levels of some hypothalamic neuropeptides, including ghrelin and adiponectin, which act upstream of kisspeptin and GnRH neurons. The present results showed that *ghrelin* gene expression levels significantly suppressed following the central injection of serotonin. The results are in line with the effects of consuming the drugs, which increase serotonin secretion such as fenfluramine and m-chlorophenylpiperazine and the drugs, which inhibit the serotonin reuptake [17-19]. It has been revealed that these drugs play a crucial role in decreasing plasma ghrelin concentration and food intakes via activating 5HT2C and 5HT1B receptor subtype of serotonin [2,17,20]. The 5HT2C receptor is expressed on pro-opiomelanocortin (POMC) neurons that are involved in producing alpha-melanocyte-stimulating hormone (αMSH), which it turn is an important stimulatory hormone for kisspeptin synthesis and GnRH/LH release [21]. Also, it has been shown that serotonin increases the production of αMSH via binding to 5HT2C [1-2]. The 5HT1B receptor is expressed on neuropeptide Y(NPY)/agoti- related peptide(AgRP) neurons of the ARC, and synthesis of these peptides is suppressed by binding serotonergic drugs to 5HT1B [1-2]. Previous studies demonstrated a reverse relationship between αMSH and ghrelin levels and a direct relationship between NPY/ AgRP and ghrelin levels [22-23]. So inhibiting NPY/ AgRP or stimulating αMSH synthesis by serotonin may partly be a missing link for the inhibitory effects of serotonin on ghrelin secretion. Also, previous studies have shown that ghrelin decreases hypothalamic kisspeptin synthesis [24]. While kisspeptin activates αMSH and inactivates neuropeptide Y(NPY) neurons [25]. So down-regulation of ghrelin synthesis following the injection of serotonin might be a possible mechanism for stimulatory effects of the serotoninergic pathway on hypothalamic *KiSS1* gene expression. Also, the present results showed that serotonin suppressed the hypothalamic *adiponectin *gene expression levels. The present results are in agreement with previous studies that demonstrated the inhibitory effects of serotonin on adiponectin hormone secretion in male rats [26]. It has been revealed that the injection of serotonin receptor antagonists, including the 5HT2 receptor, significantly increases adiponectin concentration in type 2 diabetes, which is accompanied by decreased plasma levels of adiponectin [27]. Adiponectin receptors are expressed in hypothalamic nuclei involved in the regulation of reproduction, and their expression is reported on GnRH neuron, GTI-7 cell line, and lactotroph cells of pituitary [28]. Intracerebroventricular injection of adiponectin inhibits GnRH/ LH release via activating AMP-activated protein kinase (AMPK) pathway signaling pathway [29]. Also, there is a negative correlation between adiponectin and kisspeptin signaling pathway so that increased level of adiponectin results in a significant decrease of kisspeptin synthesis [26] or decreased adiponectin levels is associated to increased levels of kisspeptin synthesis in polycystic ovary syndrome and following increased LH/FSH ratio [30]. Based on previous studies mentioned above and the results of our lab, inhibition of the synthesis of hypothalamic adiponectin following injection of serotonin may be partly involved in stimulatory effects of the serotoninergic pathway on hypothalamic *KiSS1* gene expression and following increased serum LH concentration.

## Conclusion

The intra-cerebroventricular injections of serotonin hydrochloride significantly increased the mean serum LH concentration and hypothalamic *KiSS1* gene expression levels compared to saline receiving rats. Serotonin hydrochloride significantly decreased the mean hypothalamic *ghrelin and adiponectin* gene expression levels compared to the saline group. The serotonergic pathway may exert stimulatory effects on hypothalamic kisspeptin synthesis, partly via inhibiting hypothalamic ghrelin and adiponectin neural activity.

## Acknowledgment

 The authors appreciate to Dr. Homayoun Khazali from Shahid Beheshti University for supplying the instruments.

## Conflict of Interest

 There is no conflict of interest in this article.
